# Supplementary material for: ABO Blood Groups in Systemic Sclerosis: Distribution and Association with This Disease’s Characteristics
Source: J Clin Med. 2022 Dec 24;12(1):148. doi: 10.3390/jcm12010148 (PMC9821217; doi:10.3390/jcm12010148)
Supplement: Supplementary file 1 [file jcm-12-00148-s001.zip › jcm-2106828-supplementary.pdf]

Supplementary data

Table S1: French general population and SSc population phenotypical ABO blood group repartition

	SSc population - n (%) N = 504	French general population - % (1)	p
O	210 (41.6)	43.1	0.06
A	208 (41.3)	41.9	
B	59 (11.7)	10.9	
AB	27 (5.4)	4.1	

SSc: systemic sclerosis

Table S2: French general population and SSc population O / non-O blood group repartition

	SSc population - n (%) N = 504	French general population - % (1)	p
O	210 (41.6)	43.1	0.18
Non-O	294 (58.4)	56.9	

SSc: systemic sclerosis

Table S3: General population and SSc population simplified diplotype blood group repartition

	SSc population - n (%) N = 131 diplotypes	General population - % (2)	p
AO	45 (34.35)	39.05	0.36
BO	15 (11.45)	5.9	
OO	57 (43.51)	43.79	
Other	14 (10.69)	11.24	
AA	6 (4.58)	8.28	
AB	8 (6.11)	2.38	
BB	0 (0.00)	0.58	

SSc: systemic sclerosis

Table S4: General population and SSc population diplotype blood group repartition

	SSc population - n (%)<br>N = 131 diplotypes	General population - % (2)	p
A1O1	35 (26.72)	28.74	
A2O1	9 (6.87)	10.18	
O1B	14 (10.69)	4.79	
O1O1	57 (43.51)	41.92	
Other	16 (12.21)	14.37	
A1A1	5 (3.82)	4.73	0.31
A1A2	1 (0.76)	3.55	
A1O2	0 (0.00)	0.00	
A2O2	1 (0.76)	0.59	
A1B	6 (4.58)	1.18	
A2B	2 (1.53)	1.18	
O2B	1 (0.76)	1.18	
BB	0 (0.00)	0.59	
O1O2	0 (0.00)	2.37	

SSc: systemic sclerosis

Table S5: General population and SSc population haplotype blood group repartition

	SSc population - n (%)<br>N = 262 haplotypes	General population - % (3)	p
A1	52 (19.85)	22.50	
B	23 (8.78)	5.80	
O1	172 (65.65)	61.60	0.01
Other	15 (5.73)	10.10	
A2	13 (4.96)	6.1	
O2	2 (0.76)	2.9	
Other	0 (0.00)	1.1	

SSc: systemic sclerosis

1. Bailly P, Chiaroni J, Roubinet F, Etablissement français du sang. Les groupes sanguins érythrocytaires. Montrouge; [La Plaine Saint-Denis: J. Libbey ; Établissement Français du Sang; 2015.

2. Nishimukai H, Okiura T, Shinomiya T, Fukumori Y, Ohnoki S, Shibata H, et al. Genotyping of the ABO blood group system: analysis of nucleotide position 802 by PCR-RFLP and the distribution of ABO genotypes in a German population. Int J Leg Med. oct 1996;109(2):90-3.

3. Germain M, Chasman DI, de Haan H, Tang W, Lindström S, Weng LC, et al. Meta-analysis of 65,734 Individuals Identifies TSPAN15 and SLC44A2 as Two Susceptibility Loci for Venous Thromboembolism. The American Journal of Human Genetics. avr 2015;96(4):532-42.
